# Impact of Soft Drink Intake on Bone Development and Risk of Fractures in a Danish Cohort of Schoolchildren

**DOI:** 10.3390/children12010043

**Published:** 2024-12-30

**Authors:** Helene Hermansen, Mina Nicole Händel, Malene Søborg Heidemann, Niels Wedderkopp

**Affiliations:** 1 Odense University Hospital, 5000 Odense C, Denmark; 202303533@post.au.dk; 2 Parker Institute, Bispebjerg and Frederiksberg Hospital, 2000 Frederiksberg, Denmark; mhandel@health.sdu.dk; 3 Institute of Clinical Research, Odense Patient Data Explorative Network, University of Southern Denmark, 5000 Odense C, Denmark; 4 Hans Christian Andersen Children’s Hospital, Odense University Hospital, 5000 Odense C, Denmark; malene.soborg.heidemann@rsyd.dk; 5 Research Unit of Pediatrics, Department of Clinical Research, Faculty of Health Sciences, University of Southern Denmark, 5000 Odense C, Denmark

**Keywords:** soft drinks, bone health, physical education, fracture risk, BMD, longitudinal study

## Abstract

Background and Aims: Soft drink consumption is suspected to negatively impact bone health in children, but longitudinal evidence is limited. This study assessed the association between soft drink intake and bone health outcomes in Danish schoolchildren aged 7.7–12 years, within a physical activity intervention framework. Methods: This study was nested in the CHAMPS-DK trial, a quasi-experimental study. Participants (*n* = 529) were recruited from intervention schools offering 270 min of physical education (PE) per week (active arm) and control schools with 90 min of standard PE. Soft drink intake was assessed via a food-frequency questionnaire at baseline. Dual-energy X-ray absorptiometry (DXA) was used to measure Bone Mineral Content (BMC), Bone Area (BA), and Bone Mineral Density (BMD) at baseline and two-year follow-up (primary outcomes). Fracture incidence over a five-year period was recorded using the SMS-Track parental reporting system (secondary outcome). Multilevel mixed-effects linear regression and Weibull survival models were used to analyze associations. Results: Soft drink intake of more than twice per month did not significantly affect BMC, BA, or BMD over two years (Total body BMD: *β* = 0.004; 95% CI: (−0.007; 0.016). Adjustment for confounders such as age, sex, BMI, pubertal status, socioeconomic status, and physical activity did not change the results. Additionally, no significant difference in fracture risk was observed (HR = 0.86; 95% CI: [0.43; 1.71]). Conclusions: Soft drink intake had no measurable impact on bone health indices or fracture risk in children, irrespective of PE intervention. Future studies should investigate the effects of specific soft drink types (carbonated vs. non-carbonated) on bone development.

## 1. Introduction

Bone health in childhood is determined by various factors such as gender, lifestyle, genetics, nutrition, and several diseases. Modifiable lifestyle choices made during childhood and adolescence like sedentary behaviour, excessive consumption of caffeine or beverages, malnutrition, smoking, high alcohol intake, and exposure to environmental pollutants can negatively influence bone health and may speed up the natural and gradual process of bone loss later in life [1,2].

There has been an increase in the proportion of people consuming beverages as their major source of sugar [3,4,5,6]. Sugar sweetened beverages (SSB) include, but are not limited to, all non-alcoholic, carbonated and non-carbonated beverages with added sugar during processing, e.g., soft drinks, juice, ice-tea, energy drinks and sports drinks. In recent studies, systematic reviews and meta-analysis, SSB, especially those containing phosphoric acid and caffeine, have been associated with potential negative impacts on bone health and development like lower bone mass density (BMD) and heightened risk of fractures in children and adolescents [7,8,9,10,11]. However, a cross-sectional study by Bragança found no association between SSB consumption and total BMD [12].

The mechanism behind this is partially attributed to an increased urinary excretion of calcium, which can lead to a negative calcium balance and affect bone density, caused by excessive caffeine and phosphoric acid intake [7,13]. The sugar content in beverages is also of concern since it has been linked to various health issues, including obesity. Obesity, in turn, can place additional stress on the bones and joints, potentially affecting skeletal development and bone health by elevating BMD and creating a higher risk of fractures and insulin resistance, which have a negative impact on bones [14,15,16,17,18].

Recent studies on the effects of SSB on bones have primarily been conducted outside of Europe and often utilize an observational case-control design, focusing on adults or high-risk groups with diverse covariates. Therefore, the aim of the study was the following: To assess if intake of soft drinks is associated with change in BMD, Bone Mineral Content (BMC) and Bone Area (BA) over a two-year period in healthy Danish children aged 7.7–12 years at baseline. A subsequent aim was to investigate the time to first fracture over a five-year period considering soft drink consumption as a potential risk factor.

## 2. Materials and Methods

### 2.1. Study Design and Sample

The population for this prospective cohort study comprised all students from the second, third, and fourth grades at baseline, nested within the Childhood Health, Activity, and Motor Performance School Study Denmark (The CHAMPS study). The CHAMPS study is performed in the municipality of Svendborg, which is situated in the Region of Southern Denmark on the southeast coast of the island of Funen. The municipality has approximately 60,000 inhabitants. The city of Svendborg, which has 28,000 inhabitants, is the seat of Svendborg Municipality and is a small city surrounded by a rural area with small towns and farms. The CHAMPS study is an ongoing cohort study that follows up on a natural experiment investigating the influence of attending sports schools compared to a regular public school on various health parameters. In the present study, participating children from both sports schools and traditional schools were combined into a common cohort. The CHAMPS study, including study design, eligibility criteria and methodology has been described elsewhere [19].

**Figure 1:**
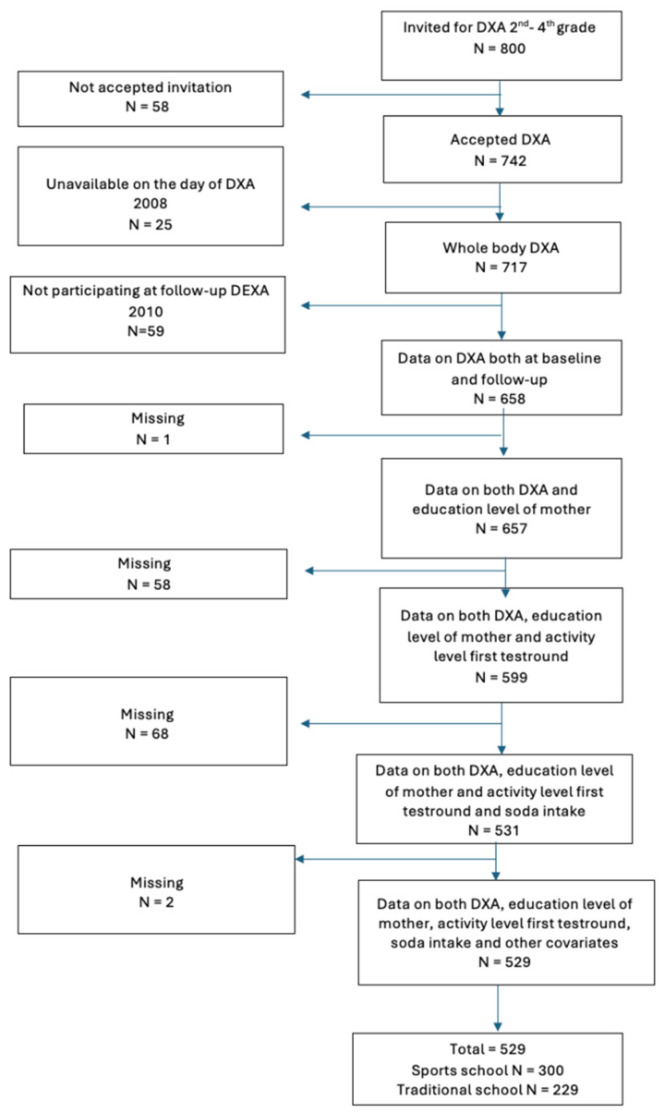
Flowchart of participants included in the analysis.

The data collection started in 2008, and follow-up data used in this study were collected two years after the baseline measurements and participants attending both these two data collection points were included in the study. Children attending 2nd to 4th grade (7.7–12 years) at baseline were invited to the first Dual Energy X-ray Absorptiometry (DXA) scan. Of the 800 children invited, 742 (93%) agreed to participate. At baseline, 717 children (97% of those enrolled) completed assessments, and 658 (89%) participated in both the baseline and follow-up DXA scans conducted two years later (ages 9.8–13.8 years at follow-up). All children invited and participating in the study were healthy, meaning all children with any type of disease at the day of measurement were not included in the study. Information on both soft drink intake, BMD, and covariates on 529 participants were obtained for fully adjusted primary analysis (Figure 1 Flowchart).

### 2.2. Outcome

#### 2.2.1. Dual Energy X-Ray Absorptiometry—Bone Measurements

BMC (g), BA (cm^2^) and BMD (g/cm^2^) were measured by whole-body DXA scans, using a GE Lunar Prodigy with Encore software (version 12.3; GE Medical Systems, Madison, WI, USA) performed at Hans Christian Andersen Children’s Hospital, Odense University Hospital, Denmark. Participants underwent scanning while lying in a supine position and dressed in undergarments, stockings, a thin T-shirt, and with a thin blanket covering them. The scanner automatically adjusted the scan depth based on the child’s age, height, and weight. Every morning, the scanner was reset, following the manufacturer’s recommendations and standardized procedures. For children and adolescents with mean age 11.4 (5–17 years), The GE Lunar Prodigy has reproducibility with precision errors (1 SD) of approximately 0.75% CV (Coefficient of Variation) for bone mass [20,21].

#### 2.2.2. Fractures

Injury data were collected weekly over 5 school years using SMS-track (web-based program, using SMS, Short-Message-Service), that enables use of text messages as a mean to perform surveys [22], with data collection paused during the six-week summer holiday and the one-week Christmas holiday. Telephonic follow-up for children reporting pain was conducted by four clinicians. If pain were still present at the telephonic contact clinical examinations and diagnoses were carried out by clinicians within two weeks at the child’s school. A standard medical record and a standardized questionnaire were completed for research purposes. If necessary, children were referred to a sports medicine clinic for further examination by an orthopedic surgeon specializing in sport injuries. The child would be sent to further para-clinical examinations, such as x-ray, ultrasound, or Magnetic resonance imaging (MRI) scan, if needed.

Information on children who were seen or treated elsewhere, e.g., during summer and Christmas break or after acute traumatic injuries, at emergency departments or by general practitioners, was collected concurrently to ensure complete data on injuries. Injuries were not recorded as new if they were identified as exacerbations of non-recovered index injuries. Diagnoses were made using the International Classification of Diseases (ICD-10), and injuries were classified based on whether they were a traumatic or an overuse injury [23].

Follow-up time in analysis for fractures for each participant began at baseline and ended at first fracture event, death, emigration, or end of study period (1 January 2015), whichever came first.

### 2.3. Soft Drink Intake

Parents were asked at baseline to complete a questionnaire with general information, including a modified food frequency questionnaire on behalf of the child. These types of questionnaires have been extensively validated in the Danish population [24,25]. They were asked “how often do you drink soft drinks? (Cola or Fanta e.g.)”, and the reply options were (1) every day, (2) almost every day, (3) between one and two times per week, (4) between one or two times per month, and (5) almost never or never. In the analysis, the exposure was dichotomized: 0 = 4–5; soft drink intake less than two times per month, 1 = 1–3; soft drink intake from every day to 1–2 times per week. The dichotomization was performed to attain statistical power as the groups got to be too small if not dichotomized. Therefore, the exposure solemnly focuses on diet and non-diet carbonated sodas, which excludes juices, energy drinks and sports drinks.

### 2.4. Covariates

To identify potential confounders for which to adjust for in analysis, a Directed Acyclic Diagram (DAG) was drawn (see Supplementary Materials). The adjustment variables chosen on basis of the DAG were age, sex (female; male), self-assessed puberty level (Tanner stage 1; 2; 3, 4, 5); mothers socioeconomic status (SES) using the educational level of the mother as proxy (1: vocational; bachelor or equivalent; master or equivalent, 2 = high school; short tertiary, 3 = 9th grade or less), number of physical education (PE) lessons (sport school = six weekly lessons; traditional school = two weekly lessons) and Body Mass Index (BMI). BMI is a suspected mediator due to high soft drink intake being associated with increase in BMI which, in turn, is linked to a high BMD [14,15,16]. In that case, adjusting for this variable would remove the association between soft drink intake and BMD.

Data on mothers’ education levels were collected at baseline. In analyses it was considered valid if an answer was given at any of respective data-collection points as the mother’s highest attained education level was not considered likely to change within the study period. Data on age and sex were collected at baseline and at the follow up DXA scan. Information on puberty level, height and weight were also gathered at these occasions. The anthropometric measures were measured with height to the nearest 0.5, and bodyweight to the nearest 0.1 kg. Children were required to go barefoot and wore only underwear and a thin t-shirt during measurement. A portable stadiometer, SECA 214, was used for measuring height and an electronic scale, SECA 861, was used for measuring weight (both from Seca Corporation, Hannover, MD, USA) [21]. BMI was calculated as weight in kg divided by height in meters squared.

### 2.5. Accelerometer

Physical activity (PA) was monitored using the Actigraph GT3X, a small triaxial accelerometer designed to measure human movement and estimate energy expenditure. This device captures movement along three planes: side-to-side (*z*-axis), front-to-back (*x*-axis), and up-and-down (*y*-axis). For the purposes of this study, only the vertical axis data were analyzed, as they are well-validated and extensively documented [21,26,27]. During the longitudinal study multiple PA assessments were conducted. However, this study focused solely on data collected between November 2009 and January 2010 where children attended 3rd to 5th grade.

The accelerometer data (movement counts) were digitalized and filtered with band limits of 0.25–2.5 Hz to remove non-relevant accelerations like vibrations. Data were recorded in 2-s intervals. Researchers distributed the accelerometers directly to the children at their schools, ensuring that children knew how to attach the device properly, placing it at the right hip using customized elastic belts, providing both verbal and written instructions to the children and their parents. The children were instructed to wear the device from the time they woke up until bedtime for seven consecutive days, including both weekdays and weekends, except during activities like showering or swimming to avoid damaging the device. After the monitoring period, the accelerometers were collected, and the data were downloaded to a computer [19]. A seven-day monitoring period is consistent with recommendations indicating that this duration is necessary to accurately capture a child’s habitual PA behavior [28]. This also argues for the validity of including only PA measures from the first test round. To strengthen the assumption of the data showing the child’s habitual PA behavior, participants with at least 10 h per day for four-or more days measurement period of PA level were included in the analysis.

### 2.6. Ethics Approval

The study was performed in accordance with the Declaration of Helsinki, approved by the Regional Scientific Ethical Committee of Southern Denmark (ID S-20080047), and registered with the Danish Data Protection Agency (J.nr. 2008-41-2240). All children gave verbal assent, and all parents or guardians provided written informed consent to participate before study enrollment.

### 2.7. Use of Chat 4o AI

We used Chat 4.o to check spelling and test for orthographic and grammatical errors in the manuscript.

## 3. Statistical Analysis

Analysis of data were performed using the STATA^®^ 18.0 software (StataCorp, College Station, TX, USA). In the descriptive analysis, continuous variables were reported with mean and standard deviations (SD) if normally distributed and with medians and interquartile ranges (IQR) if non-normal distributed. Model assumptions of normality of the residuals were checked by histograms and q–q plots. There was no indication of deviation from normality. Categorical data were reported with absolute and relative frequencies.

A sample size analysis using the variable with the highest variation (physical activity measured using accelerometers) was performed at the start of the study (19), here it was found that “with the assumption of a change of 50 counts per minute, a standard deviation of 200. With three measurements on each subject, 88 subjects in each group (a total of 176 subjects) would be needed to show a difference without taking the cluster effect into account. With an assumed cluster factor of 3, 528 subjects would be needed for these analyses”. As the variation in longitudinal DXA measurements are substantially smaller than that for physical activity measurements, we estimated that we would have sufficient statistical power to perform the study.

A multilevel mixed-effects linear model was used to analyze the association between soft drink intake and change in BMC, BA and BMD. A multilevel mixed-effects parametric survival model with Weibull distribution was used to evaluate the association between soft drink intake and risk of first fracture event. All analyses were performed unadjusted and, in three different adjustment models: First, adjusting for the evaluated bone measurement at baseline, sex, age, SES, and weekly number of PE-lessons. By adjusting for baseline bone measurements, the children’s initial size was controlled for. To avoid possible size-related artifacts in the analysis of bone mineral data, BMC was adjusted for BA at follow-up [29]. Furthermore, a second model further adjusted for BMI and pubertal level at baseline and the third model also adjusted for time spent in moderate and vigorous activity for all associations analyzed. Physical intensities were categorized according to Evenson et al. [26].

In all models’ classes and schools were included as random effects in the analyses of the association between soft drink intake and BMD, BMC, and BA at follow-up, respectively. In the cox proportional hazard models’ class was included as the random effect. By taking this approach the suspected lack of independence in the population, meaning that some of the participants attend the same class and that some of these classes belong to same schools was addressed. This is due to the multilevel modelling allowing for explicit clustering and models the relationship between dependent and independent data when there is a correlation between observations. The effect size of the associations is given through β-coefficients and hazard ratios, respectively, as well as 95% confidence intervals.

## 4. Results

More than half of the participants had a soft drink intake of more than two times per month (58.41%) the distribution is shown in Table 1.

The study characteristics are outlined in Table 2. At baseline the study population consisted of 50.47% girls. The mean age was 9.58 years, and 41.97% had a mother belonging to socioeconomic group 1 (highest attained educational level vocational; bachelor or equivalent; master or equivalent).

Baseline mean BMD was 0.76 g/cm^2^, mean BMC was 857.10 g and mean BA was 1118.37 cm^2^. During the study period 34 fractures occurred. There was 56.71% of the study population who attended sports school, 63.52% were prepubertal (Tanner stage 1) and baseline mean BMI was 16.62 (kg/m^2^).

### 4.1. Association Between Soft Drink Intake and BMD, BMC, and BA

Table 3 presents the association between soft drink intake and subsequent change in bone measurements. No evidence was found of an association between children who had a soft drink intake of more than two times per month and change in BMD over two years compared to those, who had a soft drink intake less than two times per month (β = 0.004; 95% CI: [−0.007; 0.016]). The results did not change after adjusting for confounding variables in Model 1 (β = 0.002; 95% CI: [−0.002; 0.007]), Model 2 (β = 0.003; 95% CI: [−0.001; 0.007]) or Model 3 (β = 0.003; 95% CI: [−0.001; 0.007]). Similarly, there was no association between soft drink intake and BMC or BA, respectively, in either the crude or adjusted models. Age at follow-up, sex and BMI, pubertal stage and the relevant bone measurement at baseline were predictors for BMD, BMC, and BA at follow-up in Model 3.

Membership of the clusters school and class had a significant influence on the results (*p* < 0.0001) with an intraclass correlation of 0.252, 0.302 and 0.296 for class clusters in BMD, BMC, and BA at follow-up.

### 4.2. Association Between Soft Drink Intake and Risk of Fracture

Table 4 presents the association between soft drink intake and risk of fractures. No association was found between the children who had a soft drink intake of more than two times per month and risk of first fracture compared to those, who had a less frequent intake of soft drinks than two times per month in either the crude or adjusted models (HRcrude **=** 0.86; 95% [CI: 0.43; 1.71]).

## 5. Discussion

Overall, no association was found between soft drink intake and change in BMC, BA or BMD over a two-year period in children aged 7.7–12 years at baseline or risk of first fracture over a five-year period. This finding was consistent in both the crude and multivariable models.

Two systematic reviews have previously reviewed the literature on the association between sugar-sweetened beverage intake and bone health in children and adolescents [7,10]. The outcome of interest of the first review was solemnly on fracture risk in children excluding cross-sectional studies [10]. The authors identified three case-control studies, and a meta-analysis was not feasible, due to lack of comparable data in the primary studies. In summary, two studies found an association [30,31] and one did not [32].

The later systematic review [7] included studies on BMC, BMD, and/or fracture outcomes in children with no restriction on study design. The authors identified eight studies with BMC or BMD outcomes in children: one randomized controlled trial (RCT) [33], three longitudinal studies [11,34,35], three case-control studies [31,32,36], and one cross-sectional study [37]. They also identified five studies with fracture outcome in children: three case-control studies (the same as identified by Händel et al. [30,31,33] and two cross-sectional studies [8,38]. Again, meta-analyses were deemed infeasible for all bone outcomes in children. In summary, six studies reported an association with BMC or BMD at various sites (whole body, upper and lower body, forearm, spine, and/or heel) [11,31,34,35,36,37], and two studies (a RCT and a case-control study) found no association with BMD (whole body, spine, and/or femur) [32,33]. For fracture outcome, the two cross-sectional studies [8,38], not included in Händel et al. [10], showed odds ratios ranging from 2.0 to 4.6 depending on sex and type of sugar sweetened beverages.

Overall, using vote counting based on the direction of results reported in the included studies of the two reviews [7,10], the results suggest that an increased intake of sugar-sweetened beverages is detrimental to bone health in children and adolescents. However, this synthesis method may have some limitations [39]. This especially because the evidence holds many uncertainties in the estimated results, due to methodological limitations of the primary studies, such as risk of residual confounding, risk of selection bias, risk of misclassification of exposure and/or outcomes, selective beverage reporting, missing data, small sample sizes (less than 400 participants in non-cross-sectional studies [3,11,30,31,32,33,34,35], risk of selective outcome reporting, inadequate statistical methods and risk of publication bias. These methodological limitations may lead to overestimating the results, and it is noteworthy that the only identified RCT (designed to indicate causality), showed no difference in whole body bone mass between children given different amounts of sugar-sweetened beverages (*p*  = 0.56) [33]. At this point, more high-quality large studies with more detailed data are needed for a definitive assessment of the association between sugar-sweetened beverages and bone health.

### Strengths and Limitations

The present study has several strengths. Compared to previous studies [7,10], our study population was of substantial size. Moreover, due to the detailed information collected at baseline we were able to control for numerous confounders, thus reducing the risk of residual confounding. The multilevel and longitudinal design strengthens the findings as well, since it handles the clustering of data due to children attending the same class and makes it possible to evaluate causality.

There are some limitations found in this study. Data on soft drink intake was based on recollection from children and helping parents when answering the questionnaire, which leads to risk of recall-bias. However, this would be a non-differential misclassification among participants affecting results equally towards the null hypothesis. Parents helped the children fill out the questionnaire, as young children might have problems reading and understanding questions, in addition to self-reported data from young children may be inaccurate [40,41]. However, this could entail uncertainty as the child might have had soft drink intake that the parents were not aware of and the children not remembering it [40,41]. Further the questionnaire was not validated on this particular population, and in reality using questionnaires in large studies should probably include a validation of the questionnaire in the population it is used on, even though this type of questionnaire has been extensively validated in the Danish population [24,25], shift in language, culture and habits over time can make these validations flawed and incorrect, invalidating the validations over time.

The effects of soft drink intake might differ due to different content of phosphoric acid and caffein, type of soft drink (e.g., diet or non-diet) or quantity, but we were not able to distinguish this in our data and only soft drink intake was investigated instead of SSB intake in general. The influence of soft drink intake on bone was investigated over a two-year period. It is possible that the impact on bone development will only become apparent after a longer period or later in life, cf. the hypotheses related to the Developmental Origins of Health and Disease theories. Evidently, there are specific critical/sensitive periods of development with rapid growth, e.g., childhood and adolescence, where adverse environmental exposures, such as sugar-sweetened beverages, in a limited time window, may lead to persistent changes in structure and function of the bone. In addition, we did not measure serum calcium which could have been affected by the SSB intake and been associated with bone development, and low serum calcium could have been an early warning sign of a coming influence on bone development. Further neither did we measure parathyroid hormone or calcium intake in the days before the DXA scans, which could also have influenced bone development.

We adjusted for the mother’s educational level, this information was attained at baseline and could change over time which probably would change the effect on the SSB intake. But as it is a reasonably short timeframe (two years) we consider the probability of a change and thereby a change in effect on SSB to be low.

Finally, in total there were only 34 events of first fracture in the study population during the study period (see Table 2). This leaves the analysis with little power and risk of type II error. There is also a risk of misclassification of outcome as it is reported by parents and not taken from medical records. However, due to the telephonic follow-up and possible clinical examination this bias is reduced. Also, fractures are rarely left untreated and are hence less likely to be underreported [10].

## 6. Conclusions

In conclusion, there was no influence on bone health (BMC, BA, and BMD at follow-up) or risk of fracture for children having a soft drink intake more than two times a month compared to children with a soft drink intake between every day and 1–2 times per week. These findings highlight the need for further investigation of the association between soft drink consumption patterns and bone development among children, potentially differentiating between carbonated and non-carbonated soft drinks.

## Figures and Tables

**Table 1:** Number and proportions of children.

Soft Drink Intake	
Answer	N (%)
Every day (%)	2 (0.38)
Almost every day (%)	25 (4.73)
Between one and two times per week (%)	282 (53.31)
Between one or two times per month (%)	147 (27.79)
Almost never or never (%)	73 (13.80)
Total	529 (100)

**Table 2:** Descriptive analysis of population at baseline.

Key Variables (Mean ± SD or n (%))	Participants at Baseline with Full Dataset (n = 529)		
	Total N = 529	Soft Drink Intake ≤ 2 Times per Month N = 220 (41.59)	Soft Drink > 2 Times per Month N = 309 (58.41)
Sex			
Boy	262 (49.53)	108 (49.09)	154 (49.84)
Girl	267 (50.47)	112 (50.91)	155 (50.16)
Type of school			
Sports school	330 (56.71)	126 (57.27)	174 (56.31)
Traditional school	229 (43.29)	94 (42.73)	135 (43.69)
Anthropometry			
Weight	32.38 ± 6.28	32.52 ± 6.82	32.28 ± 5.87
Height	139.09 ± 7.54	139.13 ± 8.14	139.05 ± 7.08
BMI	16.62 ± 2.07	16.66 ± 2.20	16.59 ± 1.98
Puberty stage			
Tanner stage 1	336 (63.52)	139 (63.18)	197 (63.75)
Tanner stage 2	177 (33.46)	74 (33.64)	103 (33.33)
Tanner stage 3	14 (2.65)	6 (2.73)	8 (2.59)
Tanner stage 4	2 (0.38)	1 (0.45)	1 (0.32)
Tanner stage 5	0 (0)	0 (0)	0
Age	9.58 ± 0.87	9.53 ± 0.89	9.62 ± 0.84
BMD (g/cm^2^)	0.76 ± 0.06	0.76 ± 0.06	0.76 ± 0.05
BA (cm^2^)	1118.37 ± 174.72	1121.22 ± 190.79	1116.33 ± 162.61
BMC (g)	857.10 ± 191.82	860.83 ± 211.01	854.44 ± 177.20
Fractures	34	19	15
Highest attained education level of mother			
1	222 (41.97)	117 (53.18)	105 (33.98)
2	111 (20.98)	33 (15.00)	78 (25.24)
3	196 (37.05)	70 (31.82)	126 (40.78)
Physical activity			
Sedentary Activity (mean %)	63.76 ± 5.52	64.033 ± 5.70	63.56 ± 5.39
Light activity (mean %)	28.28 ± 4.12	28.10 ± 4.23	28.41 ± 4.03
Moderate activity (mean %)	5.11 ± 1.43	5.06 ± 1.40	5.14 ± 1.44
Vigorous activity (mean %)	2.85 ± 1.32	2.80 ± 1.28	2.90 ± 1.34

BA, bone area; BMC, bone mineral content; BMD, bone mineral density; BMI, Body mass Index.

**Table 3:** Association between soft drink intake and subsequent change in bone measures.

Bone Measures	N	Soft Drink Intake β (95% CI)
Total body BMC		
Crude	551	5.49 (−38.29; 49.26)
Model 1 *	531	3.38 (−5.50; 12.26)
Model 2 **	529	3.74 (−5.08; 12.55)
Model 3 ***	529	3.46 (−5.34; 12.27)
Total body BA		
Crude	551	2.68 (−32.24; 37.59)
Model 1 *	531	2.41 (−10.01; 14.84)
Model 2 **	529	2.54 (−9.72; 14.80)
Model 3 ***	529	3.05 (−9.17; 15.26)
Total body BMD		
Crude	551	0.004 (−0.007; 0.016)
Model 1 *	531	0.002 (−0.002; 0.007)
Model 2 **	529	0.003 (−0.001; 0.007)
Model 3 ***	529	0.003 (−0.001; 0.007)

BA, bone area; BMC, bone mineral content; BMD, bone mineral density. Soft drink intake (Less than 2 times per month compared to reference group: more than 2 times per month). * adjusted for sex, BMC/BA/BMD at baseline, mothers SES, age and number of weekly PE lessons. ** adjusted for sex, BMC/BA/BMD at baseline, mothers SES, age, number of weekly PE lessons and BMI and puberty at baseline. *** adjusted for sex, BMC/BA/BMD at baseline, mothers SES, age, number of weekly PE lessons, BMI and puberty at baseline, mean moderate and vigorous PA%.

**Table 4:** Association between soft drink intake and risk of first fracture.

Fracture	N (Number of obs)	Soft Drink Intake HR (95% CI)
Crude	580	0.86 (0.43; 1.71)
Model 1 *	531	0.90 (0.44; 1.83)
Model 2 **	529	0.88 (0.43; 1.81)
Model 3 ***	529	0.88 (0.43; 1.80)

HR: Hazard ratio. Soft drink intake (Less than 2 times per month compared to reference group: more than 2 times per month). * adjusted for sex, BMC/BA/BMD at baseline, mothers SES, age and number of weekly PE lessons. ** adjusted for sex, BMC/BA/BMD at baseline, mothers SES, age, number of weekly PE lessons and BMI and puberty at baseline. *** adjusted for sex, BMC/BA/BMD at baseline, mothers SES, age, number of weekly PE lessons, BMI and puberty at baseline, mean moderate and vigorous PA%.

## Data Availability

Data are available from the CHAMPS Study Steering Committee upon reasonable request. Legal and ethical restrictions apply. Interested parties may contact professor Niels Wedderkopp (nwedderkopp@health.sdu.dk), and the following information will be required at the time of application: a description of how the data will be used, securely managed, and permanently deleted, in addition a data handler contract according to The General Data Protection Regulation (Regulation (EU) 2016/679) will be needed bfore data can be delivered.

## Supplementary Materials

The following supporting information can be downloaded at: https://www.mdpi.com/article/10.3390/children12010043/s1.
