# Supplementary material for: Impact of Soft Drink Intake on Bone Development and Risk of Fractures in a Danish Cohort of Schoolchildren
Source: Children (Basel). 2024 Dec 30;12(1):43. doi: 10.3390/children12010043 (PMC11763977; doi:10.3390/children12010043)
Supplement: Supplementary file 1 [file children-12-00043-s001.zip › children-3365743-supplementary.pdf]

Supplementary Materials

```
dag {
bb="0,0,1,1"
"Soft drink" [exposure,pos="0.143,0.487"]
AGE [adjusted,pos="0.630,0.163"]
BMD [outcome,pos="0.394,0.697"]
BMI [adjusted,pos="0.677,0.533"]
PA [adjusted,pos="0.418,0.081"]
Puberty [adjusted,pos="0.707,0.334"]
SES [adjusted,pos="0.206,0.147"]
Sex [adjusted,pos="0.140,0.289"]
"Soft drink" -> BMD
AGE -> "Soft drink"
AGE -> BMD
AGE -> BMI
AGE -> PA
AGE -> Puberty
BMI -> BMD
PA -> BMD
PA -> BMI
Puberty -> BMD
Puberty -> BMI
SES -> BMD
SES -> PA
Sex -> "Soft drink"
Sex -> BMD
Sex -> BMI
Sex -> PA
Sex -> Puberty
}
```

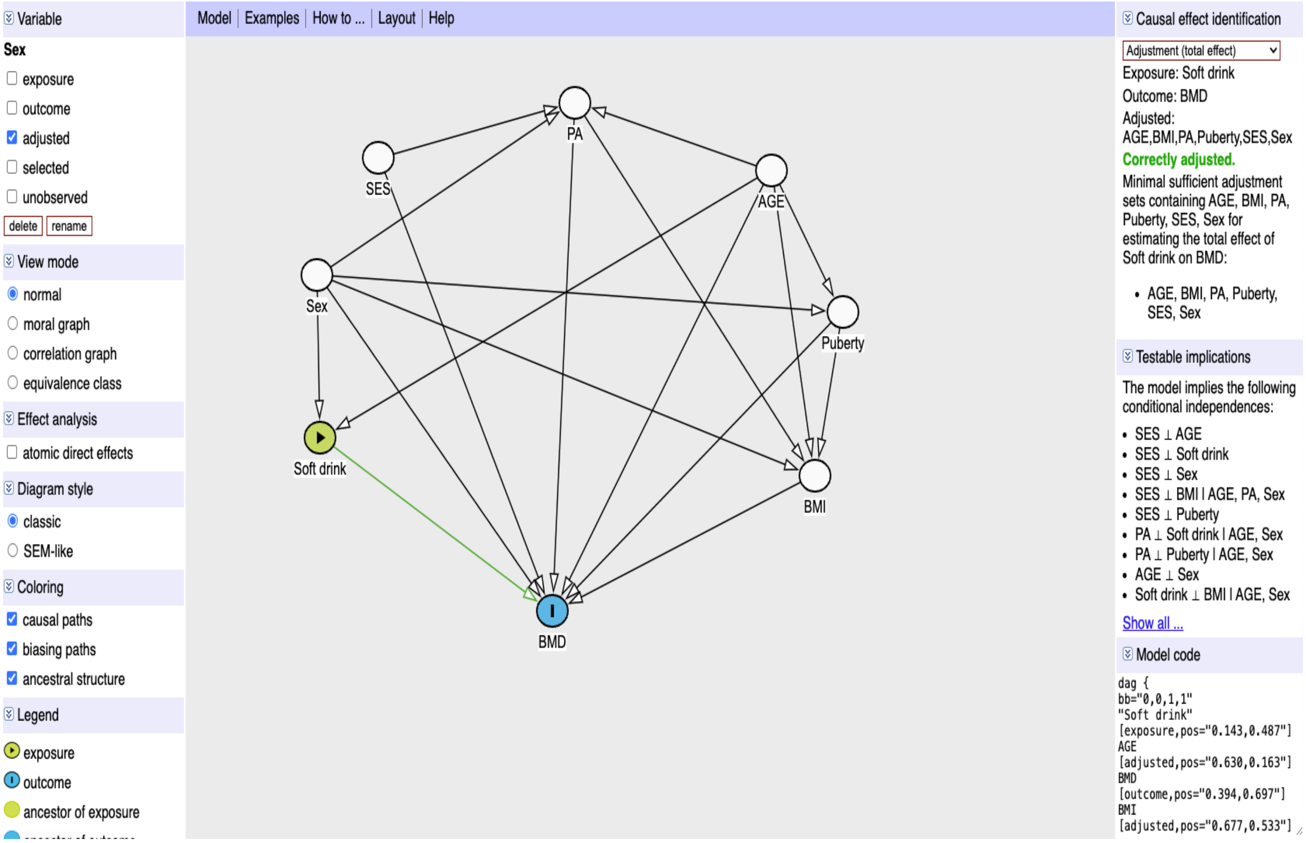
Variable
Sex
exposure
outcome
adjusted
selected
unobserved
delete
rename
View mode
normal
moral graph
correlation graph
equivalence class
Effect analysis
atomic direct effects
Diagram style
classic
SEM-like
Coloring
causal paths
biasing paths
ancestral structure
Legend
exposure
outcome
ancestor of exposure
Model
Examples
How to ...
Layout
Help
PA
SES
AGE
Sex
Puberty
Soft drink
BMI
BMD
Causal effect identification
Adjustment (total effect)
Exposure: Soft drink
Outcome: BMD
Adjusted:
AGE,BMI,PA,Puberty,SES,Sex
Correctly adjusted.
Minimal sufficient adjustment sets containing AGE, BMI, PA, Puberty, SES, Sex for estimating the total effect of Soft drink on BMD:
AGE, BMI, PA, Puberty, SES, Sex
Testable implications
The model implies the following conditional independences:
SES ⊥ AGE
SES ⊥ Soft drink
SES ⊥ Sex
SES ⊥ BMI | AGE, PA, Sex
SES ⊥ Puberty
PA ⊥ Soft drink | AGE, Sex
PA ⊥ Puberty | AGE, Sex
AGE ⊥ Sex
Soft drink ⊥ BMI | AGE, Sex
Show all ...
Model code
dag {
bb="0,0,1,1"
"Soft drink"
[exposure,pos="0.143,0.487"]
AGE
[adjusted,pos="0.630,0.163"]
BMD
[outcome,pos="0.394,0.697"]
BMI
[adjusted,pos="0.677,0.533"]
